# Highly Selective MIF Ketonase Inhibitor KRP-6 Diminishes M1 Macrophage Polarization and Metabolic Reprogramming

**DOI:** 10.3390/antiox12101790

**Published:** 2023-09-22

**Authors:** Eszter Vámos, Nikoletta Kálmán, Eva Maria Sturm, Barsha Baisakhi Nayak, Julia Teppan, Viola Bagóné Vántus, Dominika Kovács, Lilla Makszin, Tamás Loránd, Ferenc Gallyas, Balázs Radnai

**Affiliations:** 1 Department of Biochemistry and Medical Chemistry, Medical School, University of Pécs, 12 Szigeti Str., 7624 Pécs, Hungary; eszter.vamos@aok.pte.hu (E.V.); nikoletta.kalman@aok.pte.hu (N.K.); viola.vantus@aok.pte.hu (V.B.V.); dominika.kovacs@aok.pte.hu (D.K.); tamas.lorand@gmail.com (T.L.);; 2 Otto-Loewi Research Center for Vascular Biology, Immunology and Inflammation, Division of Pharmacology, Medical University of Graz, Neue Stiftingtalstraße 6, 8010 Graz, Austria; eva.sturm@medunigraz.at (E.M.S.); barsha.nayak@medunigraz.at (B.B.N.); julia.teppan@medunigraz.at (J.T.); 3 Institute of Bioanalysis, Medical School, Szentágothai Research Center, University of Pécs, 7622 Pécs, Hungary; lilla.makszin@aok.pte.hu

**Keywords:** macrophage polarization, metabolic reprogramming, glycolysis, oxidative phosphorylation, MIF, MIF inhibitor, PARP

## Abstract

Macrophage polarization is highly involved in autoimmunity. M1 polarized macrophages drive inflammation and undergo metabolic reprogramming, involving downregulation of mitochondrial energy production and acceleration of glycolysis. Macrophage migration inhibitory factor (MIF), an enigmatic tautomerase (ketonase and enolase), was discovered to regulate M1 polarization. Here, we reveal that KRP-6, a potent and highly selective MIF ketonase inhibitor, reduces MIF-induced human blood eosinophil and neutrophil migration similarly to ISO-1, the most investigated tautomerase inhibitor. We equally discovered that KRP-6 prevents M1 macrophage polarization and reduces ROS production in IFN-γ-treated cells. During metabolic reprogramming, KRP-6 improved mitochondrial bioenergetics by ameliorating basal respiration, ATP production, coupling efficiency and maximal respiration in LPS+IFN-γ-treated cells. KRP-6 also reduced glycolytic flux in M1 macrophages. Moreover, the selective MIF ketonase inhibitor attenuated LPS+IFN-γ-induced downregulation of PARP-1 and PARP-2 mRNA expression. We conclude that KRP-6 represents a promising novel therapeutic compound for autoimmune diseases, which strongly involves M1 macrophage polarization.

## 1. Introduction

Autoimmune diseases form a heterogeneous group of inflammatory conditions, which are characterized by a failure of immunological self-tolerance, resulting in a recurrent, dysregulated immune response to self-antigens [1]. Macrophage polarization is strongly involved in developing autoimmune disorders such as arthritis [2], autoimmune uveitis [3], diffuse alveolar hemorrhages caused by serious systemic lupus erythematosus [4], and many others [5]. Moreover, disturbed macrophage polarization is believed to initiate chronic inflammation [6,7]. Macrophages are mainly polarized to M1 and M2 cells. M2 macrophages are rather immunosuppressive [8] and participate in wound healing [9] or tumor formation [10]. In contrast, M1 polarized cells drive inflammation and produce high quantities of NO and pro-inflammatory cytokines such as TNF-α [11,12]. M1 macrophages undergo metabolic reprogramming characterized by a downregulation of mitochondrial energy production and accelerated glycolysis [13,14].

**Figure 1:**
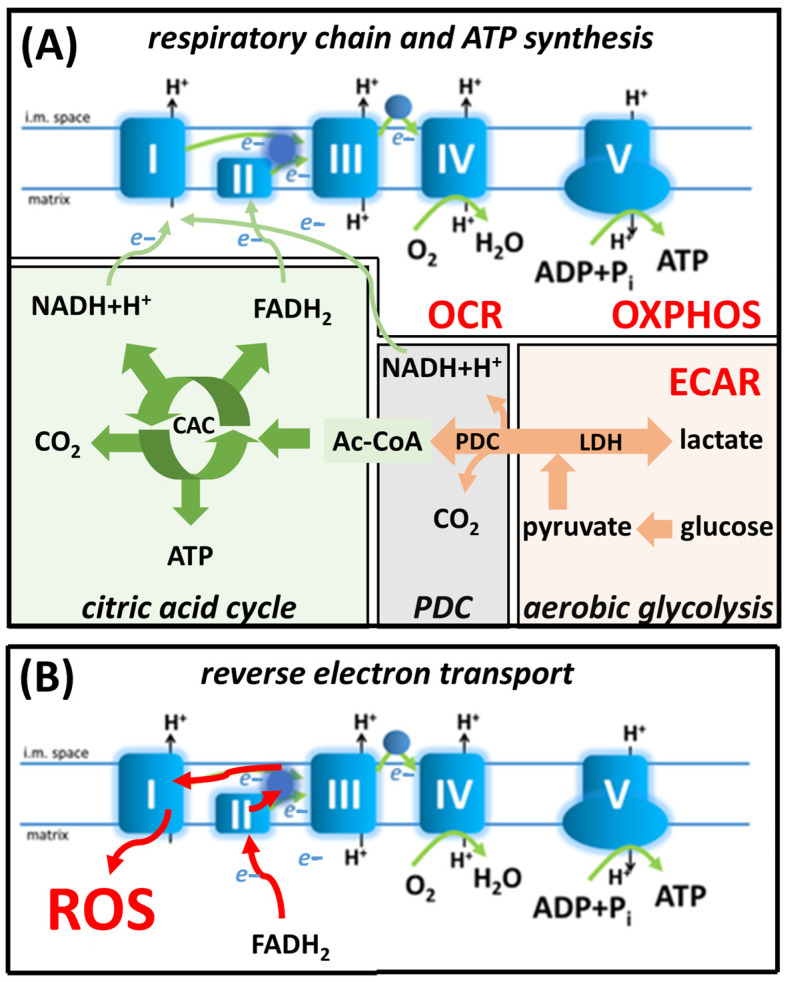
Schematic illustration of (**A**) glucose metabolism and (**B**) ROS production. Abbreviations: CAC: citric acid cycle; OCR: oxygen consumption rate; OXPHOS: oxidative phosphorylation; ECAR: extracellular acidification rate.

Mitochondrial energy production includes catabolic pathways such as the citric acid cycle (CAC). In these metabolic processes, specific dehydrogenases reduce NAD^+^ to NADH+H^+^ and FAD to FADH_2_ [15] (Figure 1). NADH and FADH_2_ provide electrons to the mitochondrial electron transport chain (mETC), thereby inducing O_2_ consumption and ATP synthesis by oxidative phosphorylation (OXPHOS) [16,17] (Figure 1).

During the metabolic switch of M1 polarized macrophages, mETC and OXPHOS become blocked [18,19] (Figure 1). To compensate for the energy loss, glucose is metabolized into lactate via aerobic glycolysis [20,21] (Figure 1A). One of the important reasons for metabolic reprogramming is to alter the role of mitochondria from ATP synthesis to ROS production [22]. M1 macrophages produce high concentrations of succinate by glutaminolysis [23,24]. During this process, glutamine is converted to 2-oxoglutarate, which is further transformed into succinate in the CAC. Elevated succinate oxidation in complex II (CII), without ATP synthesis via F_O_F_1_-ATPase, pushes the electrons in the mETC from CII to UQ, and then backwards from UQ to CI, thus inducing mitochondrial ROS production [25,26] (Figure 1B). Accordingly, classically activated M1 macrophages produce high amounts of ROS to induce “oxidative burst” [27,28]. However, “oxidative burst” is a double-edged sword: while it is highly toxic to microbes, it also leads to tissue damage in the host, and to self-induced cell death in ROS-producing macrophages. Macrophages developed various protective mechanisms against their own ROS [27]. One remarkable mechanism is the downregulation of poly(ADP-ribose) polymerase-1 (PARP-1) expression to avoid lethal PARP-1 overactivation during oxidative stress [29].

PARP-1 is a nuclear enzyme, which catalyzes the cleavage of NAD^+^ to nicotinamide and ADP-ribose. PARP-1 forms poly(ADP-ribose) (PAR) chains and PARylates numerous proteins, which leads to the activation of DNA repair enzymes [30]. Nevertheless, PARP-1 overactivation may seriously corrupt cellular energy production. On the one hand, a strong PARP-1 activation is capable of depleting NAD^+^ pools and result in mETC collapse and mitochondrial dysfunction [31]. Additionally, PAR inhibits hexokinase [32,33], the first enzyme of glycolysis, and the main energy producing metabolic pathway in M1 macrophages. Together, PARP-1 overactivation induces mitochondrial and glycolytic collapse in cells leading to strong energy depletion and cell death.

Migration inhibitory factor (MIF), the “doyen” of cytokines, was first described in the early 1950s. Many different enzyme activities of MIF have been discovered thus far, such as endonuclease [34] or the thiol-protein oxidoreductase [35]. MIF was recently implicated in the pathogenesis of several chronic inflammatory and autoimmune disease including asthma [36,37], rheumatoid arthritis, systemic lupus erythematosus [38], spinal cord injuries [39] or Hashimoto’s thyroiditis [40]. Eosinophils and neutrophils are multifunctional cells which not only contribute to first line host defense against infections, but also to initiation, modulation and resolution of inflammation, thus serving as critical effectors during inflammatory and autoimmune diseases. MIF promotes eosinophil [41] and neutrophil migration [42], and thereby contributes to accumulation of these cells leading to tissue damage and remodeling under inflammatory conditions. MIF was equally found to promote macrophage polarization towards classically activated M1 cells [43,44]. Accordingly, MIF inhibition diminishes M1 activation [45]. While the aforementioned findings raised the possibility of therapeutic utilization of MIF inhibitors during inflammatory disorders, the application was found to have limitations. The complete blockade of MIF function is rather unfavorable, since MIF has protective roles during the resolution of inflammation [46] or tissue repair [47], suggesting specific activities need to be targeted, but not MIF in general. MIF also promotes an important tautomerase activity (IUBMB Enzyme Nomenclature: EC 5.3.2.1) [48,49] by catalyzing the tautomeric keto-enol transformation of several substrates such as keto-phenyl-pyruvate to enol-phenyl-pyruvate and vice versa. Accordingly, the tautomeric activity can be divided into enolase and ketonase sub-activities [50,51].

**Figure 2:**
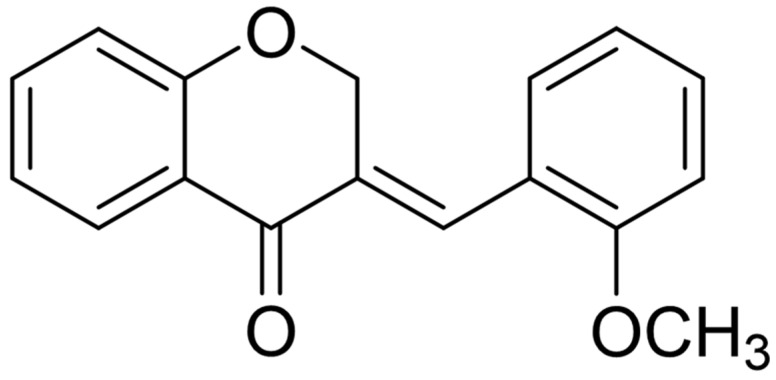
Structural formula of *E*-3-(2-methoxybenzylidene)chroman-4-one (KRP-6).

Previously, we demonstrated that E-2-arylmethylene-1-tetralones and their heteroanalogues bind the active site of MIF and inhibit MIF tautomerase activity. The best selected inhibitor of the tetralone family with both ketonase and enolase inhibitory potential repressed ROS, nitrite and cytokine production, as well as NF-κB activation in LPS-induced macrophages, and regulated thermal alterations in an experimental model of systemic inflammation [51]. In our study, we identified a potent and selective tautomerase inhibitor, KRP-6 (Figure 2), which strongly inhibited MIF’s ketonase (IC_50_ = 4.31 ± 1.34 μmol/L) but failed to reduce its enolase activity (IC_50_ = 1260 ± 159 μmol/L) [51]. In this present report, we investigated the impact of KRP-6 on M1 macrophage polarization, focusing on metabolic reprogramming and PARP mRNA transcription.

## 2. Materials and Methods

### 2.1. Test Compound

The test compound *E*-3-(2-methoxybenzylidene)chroman-4-one (KRP-6) is a known compound. It has been synthesized by a solvent-free method utilizing the 4-chromanone and the corresponding aldehyde at 140 °C with piperidine as a catalyst. The structure verification has been performed by spectroscopic methods [52].

### 2.2. Leukocyte Isolation

Assay buffer was prepared utilizing phosphate-buffered saline (PBS) supplemented with 0.9 mmol/L Ca^2+^ and 0.5 mmol/L Mg^2+^, 0.1% BSA, 10 mmol/L HEPES, and 10 mmol/L glucose, pH 7.4. PBMC spin medium was procured from pluriSelect Life Science (Leipzig, Germany). The Eosinophil Isolation Kit was received from Miltenyi Biotech (Bergisch Gladbach, Germany). (S,R)-3-(4-hydroxyphenyl)-4,5-dihydro-5-isoxazole acetic acid methyl ester (ISO-1) was procured from Merck (Vienna, Austria), MIF from Peprotech (London, UK) and IL-8 and CCL11 from Immunotools (Friesoythe, Germany). PVP-free polycarbonate filters were procured from Sterlitech (Auburn, AL, USA).

All experiments involving primary cells of human peripheral blood were approved by the Institutional Review Board of the Medical University of Graz (EK 17–291 ex 05/06). Briefly, peripheral blood polymorphonuclear leukocytes (PMNLs) were isolated from citrate-treated whole blood from healthy volunteers. Erythrocytes were removed via dextran sedimentation, and PMNLs were separated from peripheral blood mononuclear cells (PBMCs) by density gradient centrifugation utilizing PBMC spin medium (pluriSelect Life Science). Eosinophils were further separated from neutrophils by negative magnetic selection of the PMNL fraction using MACS cell separation system (Eosinophil Isolation Kit, Miltenyi Biotech), with a resulting purity of typically ≥98% [53].

### 2.3. Chemotaxis Assay

Eosinophil chemotaxis was performed with purified human eosinophils, whereas human PMNL preparations were used to assess the migratory responsiveness of neutrophils. For all experiments, technical triplicates have been performed. Cells were resuspended in assay buffer, pretreated with KPR-6 (20 µM) or ISO-1 (20 µM; Merck) for 30 min at 37 °C, and allowed to migrate towards MIF (3 nM; Peprotech; eosinophils: n = 11, neutrophils: n = 12), IL-8 (10 nM; Immunotools; neutrophils: n = 10) or CCL11 (10 nM; Immunotools; eosinophils: n = 12) for another 60 min at 37 °C in a 48-well micro-Boyden chamber using PVP-free polycarbonate filters with a pore size of 5 µm (eosinophils) or 3 µm (neutrophils) (Sterlitech). Migrated cells were enumerated by flow cytometry on a BD Canto II flow cytometer (acquisition set for 30 s at medium flow rate). Therefore, eosinophils and neutrophils were gated by their forward and side scatter properties and by autofluorescence (Figure S1) [53,54].

### 2.4. Apoptosis Assay

Isolated PMNL (n = 4) and purified eosinophils (n = 4) were pretreated with 20 µM KRP-6 for 60 min in RPMI 1640 (Fisher Scientific, Waltham, MA, USA) supplemented with 1% FBS and 1% Penicillin/Streptomycin. Next, 500 nM MIF (in PBS) was added to the cells, while PBS + BSA was utilized as a vehicle control. Following 24 h of incubation, cells were stained with APC-annexin-V (1/100) for 20 min at 4 °C and with Propidium iodide (PI; 1/50) for 1 min at room temperature in absolute darkness. Samples were immediately analyzed via BD Canto II flow cytometer (acquisition was set for 60 s at medium flow rate). The total number of live cells (annexin-V negative/PI negative), early apoptotic cells (annexin-V positive/PI negative), late apoptotic cells (annexin-V positive/PI positive) and necrotic cells (annexin-V negative/PI positive) were recorded. All experiments were executed in the form of technical triplicates [53].

### 2.5. Cell Culture and Treatments

In our cell culture experiments, we used RAW264.7 mouse monocyte/macrophage cell line (ECACC, Salisbury, UK). Cells were grown and maintained up to 10 passages in 5% CO_2_ at 37 °C in endotoxin-tested Dulbecco’s Modified Eagle’s Medium containing 4.5 g/L glucose, 2 mM L-Glutamine (Biosera), and 10% FBS (Corning) without antibiotics following thawing of frozen cells from low passage numbers. The day prior to the experiment, cells were seeded onto 96- or 24-well plates and cultured overnight. Fresh medium was added and cells were treated with 0.01 μg/mL IFN-γ (Merck, Budapest, Hungary) alone or with 0.1 μg/mL LPS from *E. coli*, 0127:B8 (Sigma-Aldrich, Budapest, Hungary) and 0.01 μg/mL IFN-γ. KRP-6 was dissolved in DMSO and applied in 20 μM concentration as a pretreatment, 30 min prior to IFN-γ alone or LPS+IFN-γ induction. To exclude the effects of vehicle, all experimental groups received the same amount of DMSO in 1:500 dilution.

### 2.6. Measurements of Free Radical Scavenging Activity

The direct free-radical scavenging activity of KRP-6 was tested in a cell free system by using the Fenton reaction [55] with 2 μM dihydrorhodamine 123 (DHR123) (Life Technologies, Carlsbad, CA, USA) fluorescent dye [56]. Oxidation of the redox dye was induced via 100 μM H_2_O_2_ and 100 μM EDTA-Fe^2+^ salt in PBS. KRP-6 was applied in 20 μM concentration in 1:500 dilution. DMSO, the vehicle for KRP-6, was applied in 1:500 dilution (0.2%), the exact same amount as with KRP-6. Fluorescent intensity of the dye (494 nm excitation and 517 nm emission) was measured immediately following the addition of DHR123 using FL6500 fluorescence spectrometer (Perkin-Elmer, Waltham, MA, USA).

### 2.7. Determination of ROS

Macrophage activation was examined by measuring reactive oxygen species (ROS) production, as previously published [49]. Shortly, RAW264.7 cells were plated onto 96-well plates at a density of 10^5^ cells/well 24 h prior to treatment. Cells were pretreated with KRP-6 (20 μM) and induced with 0.01 μg/mL IFN-γ for an additional 24 h. Next, 2 μM DHR123 [57] fluorescent dye was added, and cells were incubated for at least 2 h. Fluorescent intensity of the dye (excitation 490 nm/emission 510–570 nm) was measured with Glomax Multi Detection System (Promega^®^, Madison, WI, USA).

### 2.8. Nitrite Measurement

For nitrite measurements, we used the same culturing conditions, treatments and equipment as previously described regarding the determination of ROS. Following 24 h of incubation, 50 μL of the culture medium was added to an equal amount of Griess reagent [58] (Sigma-Aldrich) in a 96-well plate [59]. Optical density was measured at 550 nm wavelength utilizing the Glomax Multi Detection System (Promega^®^, Madison, WI, USA).

### 2.9. TNF-α Production

In consideration of TNF-α concentration measurements, RAW264.7 cells were cultured in 24-well plates at a starting density of 5 × 10^5^ cells/well and treated with KRP-6 (20 μM) for 30 min as a pretreatment together with 0.01 μg/mL IFN-γ for 24 h. TNF-α levels were determined from the culturing media via Ready-Set-Go ELISA kit (Invitrogen, Vienna, Austria). ELISA kits were applied in full accordance with the manufacturer’s protocol; absorbance was measured at 450 nm utilizing the Glomax Multi Detection System (Promega^®^, Madison, WI, USA).

### 2.10. Measurements of OCR, ECAR and Mitochondrial Bioenergetics Parameters

Mitochondrial oxygen consumption (OCR), an indicator of mitochondrial respiration, and the extracellular acidification rate (ECAR), an indicator of aerobic glycolysis of macrophages were determined by a SeahorseXFp Analyzer (Agilent Technologies, Santa Clara, CA, USA) [60,61]. The cells were seeded at a starting density of 2 × 10^4^ cells/well into Seahorse XFp Cell Culture Miniplates the day prior to treatment. The RAW264.7 cells were pretreated with 20 µM KRP-6 for 30 min and induced with LPS (0.1 μg/mL) + IFN-γ (0.01 μg/mL) for 8 h. Following pre-treatments, the medium was replaced by unbuffered Agilent XF Base assay medium pH 7.4 (serum-free), containing 10 mM glucose, 2 mM L-glutamine, and 1 mM pyruvate. The XFp Mito Stress Test Kit was utilized to evaluate mitochondrial bioenergetics. The key bioenergetic parameters were determined via specific mitochondrial respiratory chain inhibitors [62]. The applied concentration of the inhibitors was 1 μM. Bioenergetic parameters, namely basal respiration, ATP production, maximal respiration, spare respiratory capacity, non-mitochondrial respiration, proton leakage and coupling efficiency were determined by adding oligomycin, carbonyl cyanide-4(trifluoromethoxy)-phenylhydrazone (FCCP), and a mixture of rotenone and antimycin. First, oligomycin was injected as inhibitor of the F_O_F_1_-ATP synthase F_O_ subunit. ATP production was calculated based on the difference between baseline OCR and OCR following oligomycin injection. The difference between OCR after oligomycin injection and non-mitochondrial respiration revealed proton leak. The measurement of maximal respiration was accomplished by adding FCCP, which uncouples the activity of phosphorylation and oxidation. The difference between maximal and basal respiration indicated the spare respiratory capacity. Oxygen consumption following the addition of rotenone/antimycin A represented non-mitochondrial respiration. Coupling efficiency was calculated by the division of ATP production and basal respiration [62]. OCR and ECAR data were normalized to mg protein content.

### 2.11. RNA Isolation and qPCR

RAW264.7 cells were seeded on 24 well plates at a density of 5 × 10^5^ cells/well, pretreated with KRP-6 (20 µM) and cultured with LPS (0.1 μg/mL) + IFN-γ (0.01 μg/mL) for 24 h. The cells were collected, and total RNA was extracted using MagCore^®^ triXact RNA Kit (631) (RBC Bioscience Corp., New Taipei City, Taiwan) in full accordance to the manufacturer’s protocol under DNase treatment. RNA was quantified via Nanodrop 2000c spectrophotometer and Qubit 2.0 fluorometer (Thermo Fischer Scientific, Waltham, MA, USA). A total of 2 μg of total RNA was reverse-transcribed with genetically modified MMLV-based reverse transcriptase, oligo(dT) and random hexamer primers (Maxima First Strand cDNA Synthesis Kit, Thermo Fischer Scientific, Waltham, MA, USA). A total of 100 ng cDNA, together with the indicated primer pairs (Table 1) was utilized in 20 μL reactions to perform real-time PCR using Xceed qPCR SG 2× Mix (Institute of Applied Biotechnologies, Praha-Strašnice, Czech Republic) and a CFX96 Touch Real-Time PCR Detection System (Bio-Rad, Hercules, CA, USA). Data were analyzed by the ΔΔCt method. GAPDH was used as a reference for gene expression.

### 2.12. Statistical Analyses

All statistical analyses were performed utilizing SPSS version 28.0 statistics software (IBM, New York, NY, USA) or GraphPad Prism 9. First, the normality of data distribution was investigated via Q-Q plot and/or box-plot, parallel with the Shapiro–Wilk test. One-way ANOVA or Welch’s ANOVA with appropriate post hoc tests were performed to compare the means of groups. Meanwhile, the Kruskal–Wallis non-parametric one-way ANOVA was implemented in reference to independent samples with multiple pairwise comparisons, which were used to determine differences without assumption of normality. Moreover, a paired samples *t*-test was equally performed. *p*-values of less than 0.05 were considered significant.

In detail: Leukocyte recruitment, n = 10–12 independent experiments with 3 biological replicates; one-way ANOVA. Apoptosis assay, n = 4 independent experiments with 3 biological replicates; repeated-measures *t*-test. Radical scavenging, n = 10 independent experiments and Welch’s ANOVA test. Nitrite measurement, combined data of n = 18 (results of 3 independent experiments with 6 biological replicates); one-way ANOVA. ROS determination, combined data of n = 18 (results of 3 independent experiments with 6 biological replicates) and the Kruskal–Wallis test. TNF-α measurement, combined data of n = 6 (results of 3 independent experiments with 2 biological replicates); Kruskal–Wallis test. ATP production, coupling efficiency, maximal respiration, spare respiratory capacity, proton leakage, basal ECAR, combined data of n = 10 (results of 5 independent experiments with 2 biological replicates) and the Kruskal–Wallis test. Basal respiration, combined data of n = 10 (results of 5 independent experiments with 2 biological replicates) and the Welch’s ANOVA test. Extracellular acidification rate change, n = 10 (results of 5 independent experiments with 2 biological replicates); paired samples *t*-test. PARP-1,2,3 transcription, n = 6 (results of 3 independent experiments with 2 biological replicates) and one-way ANOVA.

## 3. Results

### 3.1. KRP-6 Inhibited Leukocyte Migration Similarly to ISO-1

**Figure 3:**
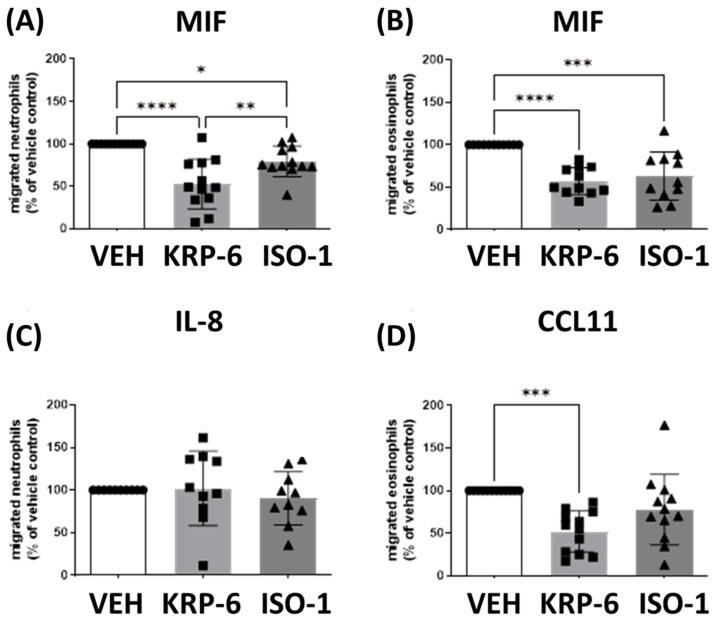
KRP-6 alleviates the migratory responsiveness of human neutrophils and eosinophils. (**A**,**C**) Polymorphonuclear leukocytes (PMNL) and (**B**,**D**) purified eosinophils were pretreated with KRP-6 (20 µM) at 37 °C for 30 min and were allowed to migrate towards (**A**,**B**) MIF (3 nM, n = 11–12), (**C**) IL-8 (10 nM, n = 10) or (**D**) CCL11 (10 nM, n = 12) in a micro-Boyden chamber at 37 °C for 60 min. Migrating cells were enumerated by flow cytometry on a BD Canto II flow cytometer (acquisition set for 30 s at medium flow rate) and expressed as % of the respective vehicle control (MIF, IL-8 or CCL11). Data are shown as mean ± SD of indicated independent experiments, all experiments were performed in the form of technical triplicates; * *p* < 0.05, ** *p* < 0.01, *** *p* < 0.001, **** *p* < 0.0001. Abbreviations: VEH: vehicle, MIF: macrophage migration inhibitory factor, CCL11: C-C Motif Chemokine Ligand 11.

MIF was shown to be upregulated during chronic inflammation and to regulate leukocyte migration by binding to cell surface receptors such as CXCR2, CXCR4 and CD74 [63]. To compare the effects of KRP-6 and ISO-1 on leukocyte migration, we isolated human peripheral blood neutrophils and eosinophils and induced migration via various chemotactic compounds. Neutrophil migration was stimulated with MIF (Figure 3A) or IL-8 (Figure 3C) and eosinophils were recruited with MIF (Figure 3B) or CCL11 (Figure 3D). Responses to chemoattractants were normalized to 100%. IL-8 and CCL11 are specific neutrophil [64] and eosinophil [65] chemotactic factors, respectively. We found that ISO-1 and KRP-6 significantly reduced MIF-induced neutrophil migration, respectively (Figure 3A). In contrast, KRP-6 and ISO-1 did not influence IL-8-stimulated chemotactic movement of neutrophils (Figure 3C). MIF (Figure 3B) and CCL11 (Figure 3D) stimulated eosinophil migration. KRP-6 and ISO-1 decreased MIF-induced eosinophil migration (Figure 3B). Interestingly, KRP-6 inhibited CCL11-induced eosinophil chemotaxis, while ISO-1 failed to initiate a decrease (Figure 3D) in a statistically significant manner.

### 3.2. KRP-6 Counteracts the Anti-Apoptotic Effect of MIF in Human Neutrophils and Eosinophils

**Figure 4:**
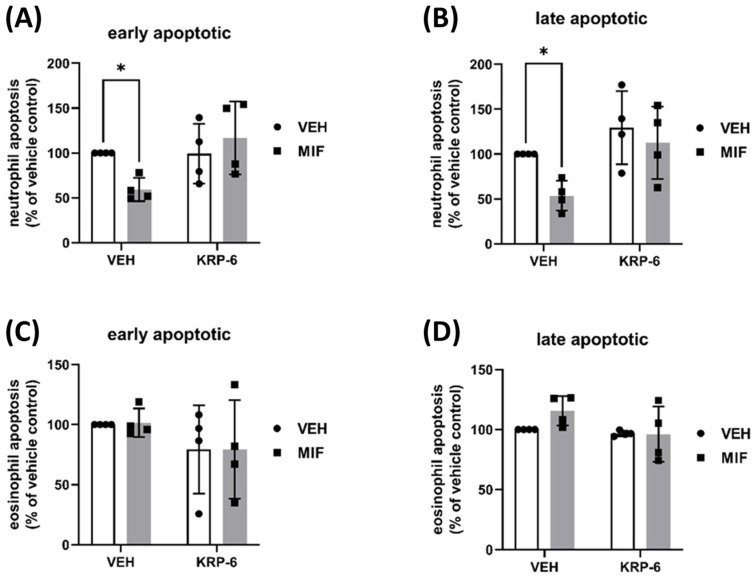
KRP-6 counteracts the anti-apoptotic effect of MIF in human neutrophils and eosinophils. (**A**,**B**) Polymorphonuclear leukocytes (PMNL, n = 4) and (**C**,**D**) purified eosinophils (n = 4) were pretreated with KRP-6 (20 µM) at 37 °C for 60 min in RPMI 1640 medium supplemented with 1% FBS and 1% Penicillin/Streptomycin. Next, MIF (500 nM), or a vehicle control (BSA) in PBS, were added to the cells. Following 24 h of incubation, cells were stained with APC-annexin-V (1/100) and Propidium iodide (1/50). Samples were immediately analyzed by flow cytometry (acquisition set for 60 s at medium flow rate). (**A**–**D**) Results of early apoptotic (annexin-V positive/PI negative) (**A**,**C**), and late apoptotic (annexin-V positive/PI positive) (**B**,**D**) cells are presented. Data are shown as the mean ± SD of all indicated independent experiments and expressed as % of the respective vehicle control. Repeated measures *t*-test, * *p* < 0.05. All experiments were performed in the form of technical triplicates. Abbreviations: VEH: vehicle, MIF: macrophage migration inhibitory factor.

Since MIF was previously shown to inhibit neutrophil apoptosis via direct and indirect mechanisms [66,67], we next examined whether KRP-6 is capable of modulating apoptosis in neutrophils and eosinophils, respectively. To the answer of our premise, isolated PMNLs or purified eosinophils were pre-treated with KRP-6 (20 µM), as described in the Materials and Methods section. Next, MIF (500 nM), or a vehicle, was added to the cells. Following 24 h of incubation, cells were stained with APC-annexin-V and with Propidium iodide and analyzed by flow cytometry. Interestingly, our results revealed that MIF attenuated early (Figure 4A) and late apoptosis (Figure 4B) in neutrophils, following 24 h of incubation; however, it did not affect eosinophil apoptosis (Figure 4A,B). Early apoptosis was reduced by 40%, whereas late apoptosis decreased by 47%. Pre-treatment with KRP-6 for 60 min prior to MIF incubation prevented anti-apoptotic effect, indicating a counteraction in one of the pro-inflammatory properties of MIF in neutrophils.

### 3.3. KRP-6 Reduces ROS in Macrophages without a Direct Antioxidant Effect

**Figure 5:**
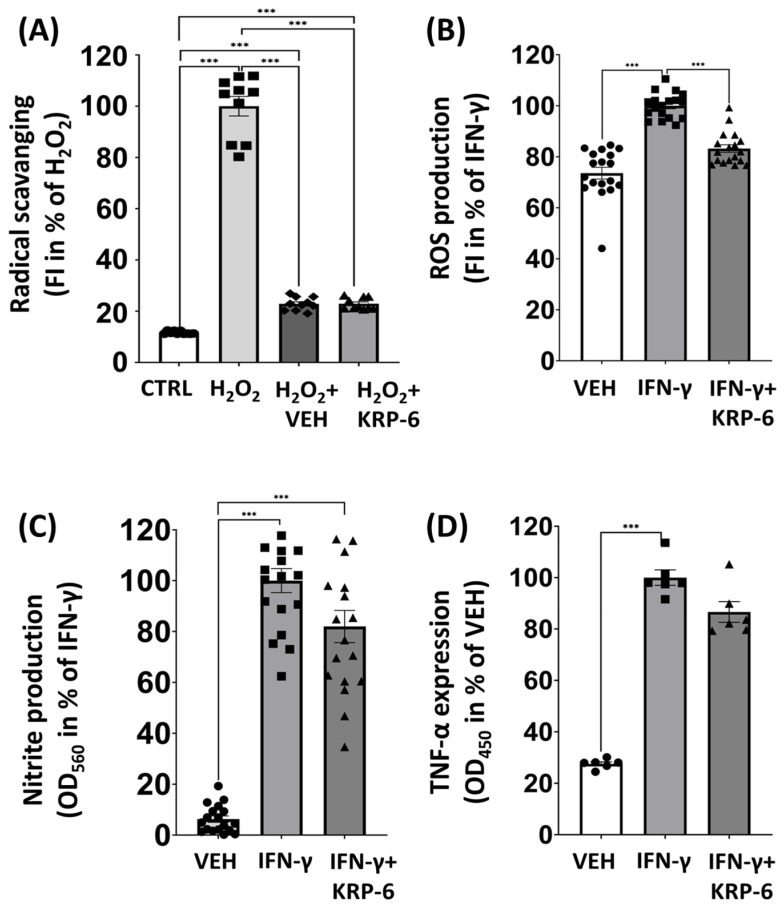
KRP-6 is definitively not a radical scavenger; however, it is capable of inhibiting ROS production in macrophages. (**A**) The concentration of peroxide radicals was measured in a cell free system by adding 100 μM H_2_O_2_ and 100 μM EDTA-Fe^2+^ salt to PBS with DMSO (vehicle for KRP-6) or KRP-6 (20 μM). The amount of DMSO was equal in the DMSO and KRP-6-treated cells. Data are expressed as means ± SEM in the percentage of H_2_O_2_. Experiments were repeated ten times (n = 10). KRP-6 was applied in 20 μM as a pretretment for 30 min and RAW264.7 macrophages were induced by 0.01 μg/mL IFN-γ for 24 h. (**B**) ROS concentration was evaluated by adding 2 μM dihydrorhodamine 123 fluorescent dye (fluorescent intensity; 490 nm [excitation]/510–570 nm [emission] wavelengths). (**C**) Nitrite production was measured using Griess reagent (optical density; 550 nm). Data of ROS and nitrite production are presented as means ± SEM in the percentage of IFN-γ-treated group (combined data of n = 18 (results of 3 independent experiments with 6 biological replicates)). (**D**) TNF-α concentrations were measured via ELISA-kits (optical density; 450 nm). Data are presented as means ± SEM in the percentage of IFN-γ-treated group (combined data of n = 6 (results of 3 independent experiments with 2 biological replicates)); *** *p* < 0.001. Abbreviations: CTRL: control, VEH: vehicle, IFN-γ: interferon-gamma.

Next, we measured the direct free-radical scavenging activity of KRP-6 in a cell free system. We equally determined ROS, nitrite and TNF-α production in IFN-γ-induced RAW264.7 macrophages (Figure 5). We found that EDTA- Fe^2+^ salt catalyzed the formation of peroxide-radicals from H_2_O_2_, which was detected through the oxidation of a DHR123 fluorescent dye. In our experiments, KRP-6 did not reduce the amount of free-radicals, i.e., it had no direct antioxidant effect. In contrast to KRP-6, the vehicle DMSO strongly inhibited peroxide production (Figure 5A). IFN-γ treatment caused higher ROS levels in macrophages when compared to vehicle-treated cells (from 73.5% to 100%). Our results equally revealed that, although KRP-6 could not directly scavenge peroxide radicals (Figure 5A), it significantly reduced ROS production to 82.3% in the IFN-γ-treated group (Figure 5B). IFN-γ-activated macrophages also produced excessive amounts of nitrite (Figure 5C) and TNF-α (Figure 5D), while KRP-6 failed to initiate a decrease in a statistically significant manner.

### 3.4. KRP-6 Reduces Glycolytic Flux in Activated Macrophages

**Figure 6:**
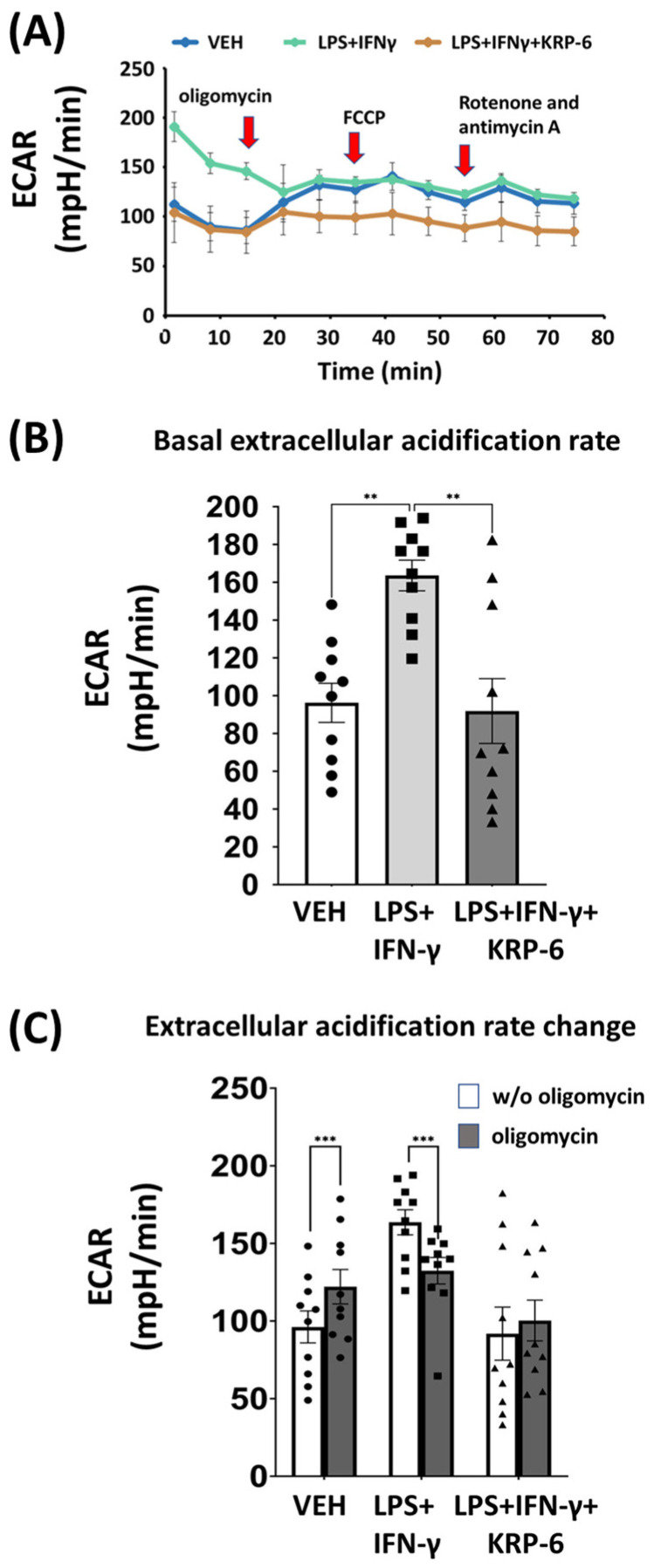
KRP-6 decreases aerobic glycolysis in activated macrophages. RAW264.7 cells were pretreated with 20 μM KRP-6 for 30 min and then induced with LPS (0.1 μg/mL) + IFN-γ (0.01 μg/mL). After 8 h following treatment, Seahorse XFp Mito Stress test was performed. VEH and LPS+IFN-γ groups received the same amount of DMSO as KRP-6-treated cells. During measurements, oligomycin, FCCP and the mixture of rotenone and antimycin A were continually added. The final concentrations of inhibitors and uncoupling agent were 1 μM. (**A**) Extracellular acidification rate (ECAR), (**B**) basal ECAR, and (**C**) ECAR alterations are shown. ECAR changes were determined by calculating the difference between ECAR values prior to and following oligomycin injection. Data are presented as means ± SD (**A**) and means ± SEM (**B**,**C**) in the percentage of LPS+IFN-γ-treated group (combined data of n = 10 (results of 5 independent experiments with 2 biological replicates)); ** *p* < 0.01, *** *p* < 0.001. Abbreviations: ECAR: extracellular acidification rate, LPS: lipopolysaccharide, IFN-γ: interferon-γ.

M1 macrophages remodel and alter their metabolism from oxidative phosphorylation to aerobic glycolysis (Figure 1). Accordingly, we determined ECAR in LPS+IFN-γ-treated RAW264.7 cells, as it represents lactate production, which refers to the activity of fermentative ATP production of the cells (i.e., aerobic glycolysis) (Figure 1 and Figure 6). To analyze the aforementioned processes, we utilized a mixture of activators (LPS+IFN-γ-treatment) to initiate the highest possible level of metabolic alterations [68].

We revealed that LPS+IFN-γ significantly enhanced the basal ECAR (without oligomycin, 1–3 points of the measurement) in macrophages when compared to vehicle-treated cells 8 h following treatment (Figure 6A,B). In contrast, KRP-6 decreased basal ECAR to the level of the VEH group (Figure 6A,B). Moreover, oligomycin treatment enhanced ECAR (4–6 points of the measurement) in VEH-treated and in LPS+IFN-γ-treated cells, yet failed to modify it in the LPS+IFN-γ+KRP-6 treatment groups (Figure 6A,C). Finally, FCCP, rotenone and antimycin A (7–9 points of the measurement) did not further modulate ECAR in any of the treatment groups (Figure 6A).

### 3.5. KRP-6 Improves Mitochondrial Respiration of Macrophages

**Figure 7:**
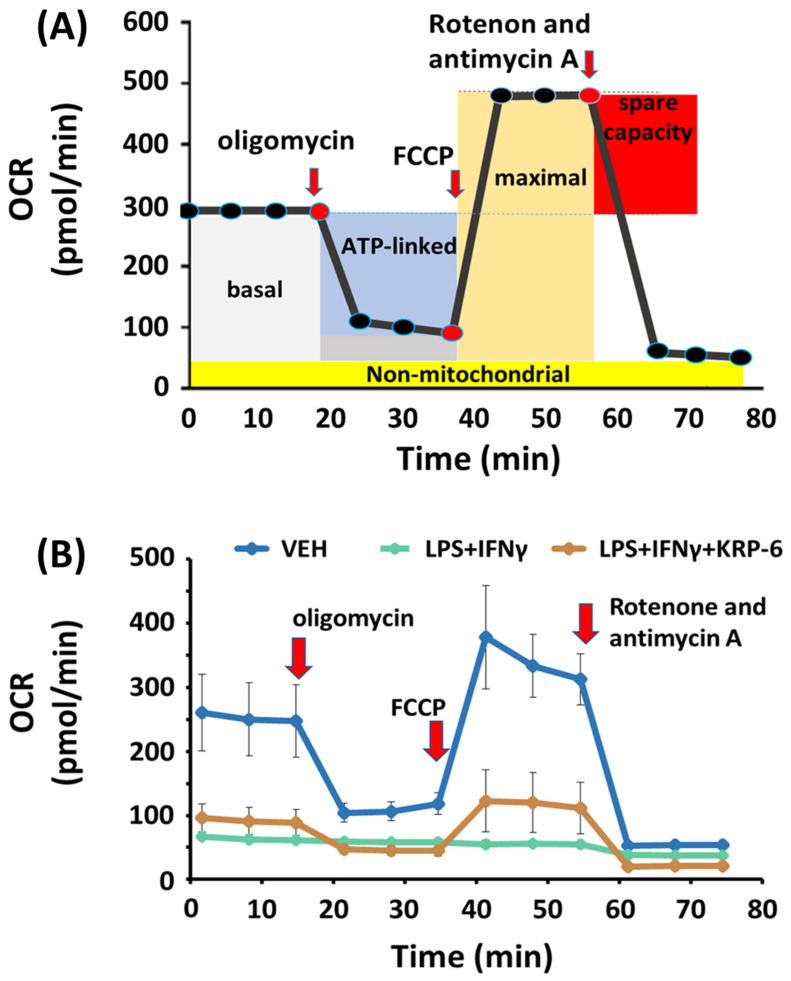
KRP-6 protects mitochondrial respiration in LPS+IFN-γ-induced macrophage cells. RAW264.7 cells were pretreated with 20 μM KRP-6 for 30 min. Then, macrophages were treated with LPS (0.1 μg/mL) + IFN-γ (0.01 μg/mL) for 8 h. VEH and LPS+IFN-γ groups received the same amount of DMSO as KRP-6-treated cells. Oligomycin, FCCP and the mixture of rotenone and antimycin A were added sequentially during the measurements in the final concentrations of 1 μM. (**A**) Key parameters of mitochondrial respiration assessed by Seahorse XFp Extracellular Flux Analyzer. (**B**) Measurement of oxygen consumption rate. Data (combined from 5 separate experiments with 2 biological replicates, n = 10) are expressed as mean ± SD. Abbreviations OCR: oxygen consumption rate, LPS: lipopolysaccharide, IFN-γ: interferon-γ.

M1 polarized macrophages downregulate oxidative phosphorylation [69]. Thus, we examined the activity of mitochondrial ETC and OXPHOS by measuring the rate of oxygen consumption in LPS+IFN-γ-induced macrophages (Figure 7).

**Figure 8:**
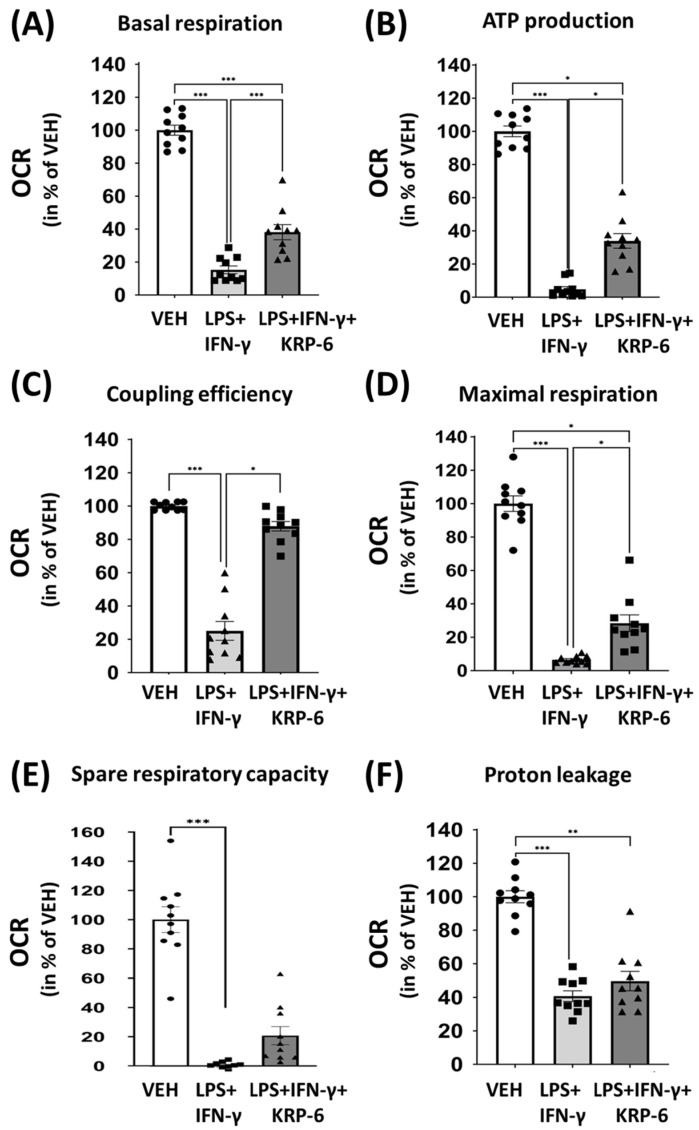
KRP-6 improves mitochondrial bioenergetic parameters in activated macrophages. RAW264.7 cells were pretreated with 20 μM KRP-6 for 30 min. Then, macrophages were treated with LPS (0.1 μg/mL) + IFN-γ (0.01 μg/mL) for 8 h. VEH and LPS+IFN-γ groups received the same amount of DMSO as KRP-6-treated cells. During measurements, oligomycin, FCCP and the mixture of rotenone and antimycin A were continuously added in the final concentrations of 1 μM. Bioenergetic parameters: (**A**) basal respiration, (**B**) ATP production, (**C**) coupling efficiency, (**D**) maximal respiration, (**E**) spare respiratory capacity, and (**F**) proton leak were determined. Results are represented in percentage of vehicle (mean ± SEM of 5 independent experiments with 2 biological replicates, n = 10); * *p* <0.05, ** *p* <0.01, *** *p* < 0.001. Abbreviations OCR: oxygen consumption rate, LPS: lipopolysaccharide, IFN-γ: interferon-γ.

First, we determined the basal OCR (basal respiration) without oligomycin treatment (grey field on Figure 7A). We found that LPS+IFN-γ initiates a significant drop, while KRP-6 significantly enhances basal respiration in comparison to the VEH treatment (1–3 points of the measurement on Figure 7B and Figure 8A).

Next, we analyzed the ATP production-linked OCR by utilizing oligomycin (blue field on Figure 7A; 4–6 points of the measurement on Figure 7B). ATP production-linked OCR refers to OXPHOS activity and ATP production in the mitochondrial F_O_F_1_-ATPase. Our results revealed that oligomycin strongly decreases OCR in the VEH and, to a lesser extent, in the LPS+IFN-γ+KRP-6 treated groups, yet failed to do so in the LPS+IFN-γ-treated cells (Figure 7B). This indicates a collapse of the mitochondrial ATP synthesis in the LPS+IFN-γ-treated cells, which can be counteracted by KRP-6 treatment (Figure 8B). In addition, LPS+IFN-γ largely decreased coupling efficiency, which was effectively increased by KRP-6 (Figure 8C).

FCCP, a mitochondrial uncoupler induces the highest level of OCR in the cells, i.e., the maximal respiration (mustard yellow field on Figure 7A). In our experiments FCCP strongly enhanced OCR in the VEH and in the LPS+IFN-γ+KRP-6 groups, yet not in LPS+IFN-γ-treated cells (7–9 points of the measurement on Figure 6B), which resulted in decreased maximal respiration in the LPS+IFN-γ group when compared to VEH. All effects were successfully reversed by KRP-6 (Figure 8D).

Rotenone and antimycin A are inhibitors of the mitochondrial respiratory chain complex I, which entirely abolishes oxygen consumption in all experimental groups (10–12 points of the measurement on Figure 7B), allowing us to determine spare respiratory capacity (red field on Figure 7A). We found LPS+IFN-γ largely abolished spare respiratory capacity, which was not modified by KRP-6 treatment (Figure 8E). Furthermore, KRP-6 did not reverse LPS+IFN-γ reduced proton leakage (Figure 8F).

### 3.6. KRP-6 Diminishes M1 Macrophage Polarization-Associated PARP-1 and PARP-2 mRNA Downregulation

**Figure 9:**
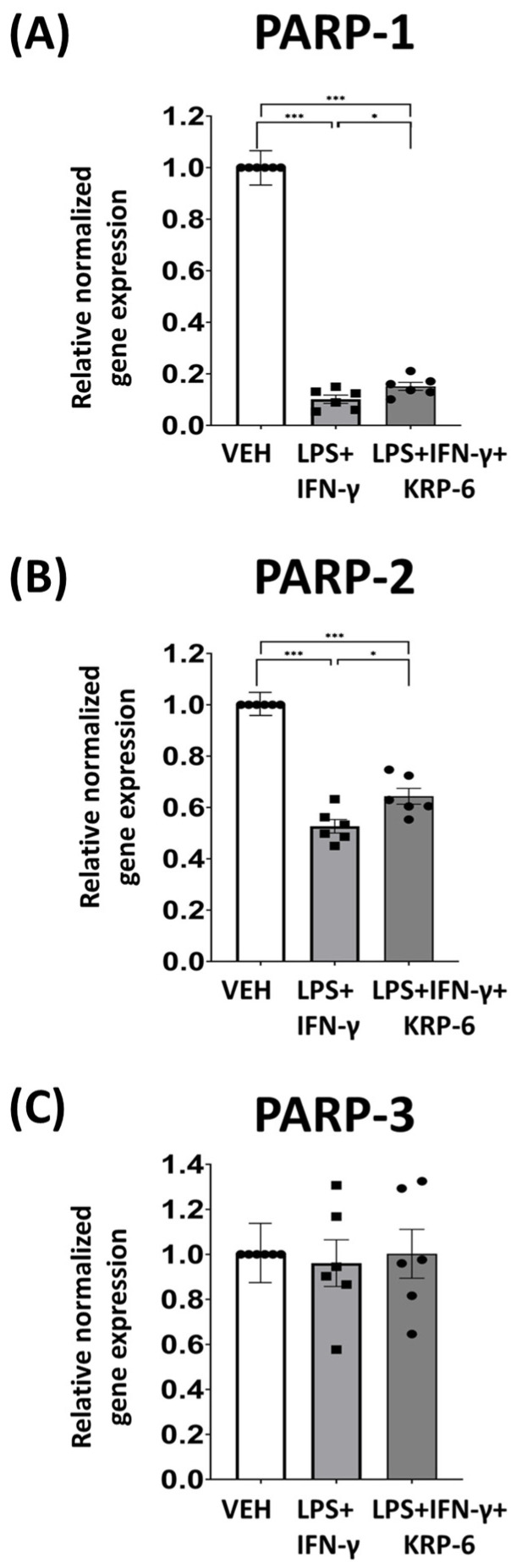
KRP-6 improves PARP-1 and PARP-2 mRNA transcription in activated macrophage cells. RAW264.7 cells were pretreated with 20 μM KRP-6 for 30 min. Then, macrophages were treated with LPS (0.1 μg/mL) + IFN-γ (0.01 μg/mL) for 24 h. VEH and LPS+IFN-γ groups received the same amount of DMSO as KRP-6-treated cells. (**A**) PARP-1, (**B**) PARP-2 and (**C**) PARP-3 relative normalized gene expressions are shown. Data (combined from three separate experiments with two biological replicates, n = 6) are expressed as mean ± SEM; * *p* <0.05, *** *p* < 0.001. Abbreviations: LPS: lipopolysaccharide, IFN-γ: interferon-γ.

Earlier results suggest that activated macrophages reduce PARP-1 mRNA transcription [29]. Accordingly, we analyzed PARP-1, -2, and -3 mRNA transcription in LPS+IFN-γ-treated RAW264.7 cells (Figure 9). Our experiments revealed that, while PARP-1 and PARP-2 mRNA transcription was reduced in LPS+IFN-γ-induced macrophages (Figure 9A,B), PARP-3 mRNA synthesis remained unaltered (Figure 9C). In contrast, KRP-6 treatment significantly enhanced PARP-1 and PARP-2 transcription (Figure 9A,B).

## 4. Discussion

The essence and goal of this present study was to investigate whether KRP-6, a potent and selective tautomerase inhibitor, may inhibit leukocyte migration via MIF binding in vitro. For this purpose, we utilized isolated human peripheral blood neutrophils and eosinophils, and induced their migration directly with MIF. Furthermore, we also investigated the impact of KRP-6 upon macrophage activation, more precisely, in the regulation of metabolic reprogramming and PARP-1,-2,-3 mRNA expression. Thus, we utilized IFN-γ- and LPS+IFN-γ-treated RAW264.7 cells, a widely accepted in vitro model, for classically activated M1 macrophages [70,71]. LPS- or IFN-γ-induced macrophages produce and secrete high amount of MIF [72,73] which makes this model most suitable for the proposed investigations regarding MIF activity.

To support the previous molecular docking analyses of KRP-6 binding to MIF [51], we first analyzed the molecule’s effect on MIF-induced leukocyte migration (Figure 3). The chemotactic response of leukocytes is thought to be initiated by binding MIF to the receptors CD74 [74,75] and CXCR2 [76]. Several lines of evidence demonstrate that receptor activation requires binding to the MIF tautomerase catalytic domain [77]; however, the tautomerase activity is not essential for receptor activation per se [78,79]. Proline to serine or proline to glycine mutant MIF molecules, which lack the catalytically essential N-terminal proline, were shown to be enzymatically inactive, yet still capable of modulating monocyte chemotaxis [80]. These results underline the premise, receptor activation requires a protein–protein interaction; however, not tautomerase activity. Therefore, we utilized a classic MIF tautomerase inhibitor, ISO-1, which was shown to bind to MIF’s tautomerase activity site [48,81,82]. ISO-1 has been also introduced to inhibit leukocyte migration [83,84]; thus, we compared its effect with that of KRP-6 (Figure 3). An existing physical interaction between KRP-6 and the MIF catalytic domain has been previously demonstrated by our research team [51]. Accordingly, the observed inhibition of MIF-induced leukocyte chemotaxis by KRP-6 can be satisfactorily explained by a physical binding to the tautomerase activity site, similarly to ISO-1. Surprisingly, KRP-6 also inhibited CCL11-induced eosinophil migration in contrary to ISO-1 (Figure 3D). The phenomenon may be explained by the existing cross-talk between MIF and CCL11 during allergic eosinophil activation [85]. Thus, neutralizing MIF receptor activation by KRP-6 may also affect CCL11 signaling in eosinophils, and thereby reducing the migratory responsiveness of the cells. Since receptor activation does not require tautomerase activity and KRP-6 inhibited eosinophil migration when ISO-1 failed to do so, KRP-6 likely has a stronger affinity for MIF than ISO-1. Thus, to achieve statistical significance, higher ISO-1 concentrations may be needed. Similar to ISO-1, KRP-6 did not modulate IL-8-induced neutrophil migration (Figure 3C). Although IL-8 and MIF share the same CXCR2 receptor [86,87], KRP-6 and ISO-1 are not CXCR2 receptor antagonists. Accordingly, neither impairs IL-8 binding or IL-8-induced neutrophil migration. Comprehensively, in consideration of these observations, KRP-6 binds to the MIF tautomerase active site more strongly than the classic tautomerase inhibitor ISO-1 in primary human eosinophils and neutrophils. Our findings suggesting that KRP-6 reduces the anti-apoptotic effect of MIF in neutrophils equally underline and support the direct binding of KRP-6 to MIF [51] (Figure 4).

Proline to alanine mutant MIF (P1A MIF) presented identical substrate affinity (Km = 310  ±  50 μM) than wild type MIF (Km = 303  ±  70 μM). In contrast to the above, the catalytic ability of mutant MIF (k_cat_ = 1.7  ±  0.3 s^−1^) decreased dramatically when compared to the wild type MIF (k_cat_ = 410  ±  50 s^−1^) [88]. Thus, P1A MIF has a structurally intact yet enzymatically inactive tautomerase active site, which binds and activates the MIF receptor, even without tautomerase activity. Interestingly, P1A MIF was unable to increase mRNA transcription of matrix-metalloprotease-1 and -3 in fibroblasts compared to wild type MIF [89]. These results emphasized the role of tautomerase activity regarding MIF’s biological functions, other than receptor activation as a cytokine. Therefore, we applied IFN-γ, a Th1 cytokine, which was demonstrated to induce MIF synthesis and secretion in RAW264.7 cells [90]. However, the autocrine receptor activation was not excluded by our experiments. As presented earlier, MIF is capable of entering the cytoplasm via receptor-mediated endocytosis and directly binding to intracellular proteins such as Jab1, a coactivator of AP-1 transcription factor [91]. Thus, a direct intracellular effect through the tautomerase activity of MIF may be achieved by this model.

Earlier results suggest that MIF induces ROS, NO and proinflammatory cytokine production, the hallmarks of inflammatory M1 macrophage polarization [92,93]. Due to this phenomenon, we analyzed ROS, nitrite (oxidation product of NO) and TNF-α production in IFN-γ-induced RAW264.7 cells following KRP-6 treatment (Figure 5B,C). Knowingly, antioxidants affect inflammatory macrophage activation by eliminating ROS and thereby lowering nitrite and TNF-α production [94]. Our present results, however, overruled this possibility (Figure 5A) and concluded that KRP-6, the selective MIF ketonase inhibitor, effectively inhibits ROS production, a hallmark of M1 macrophage polarization without a radical scavenging effect.

ECAR reflects lactate production, a measure of aerobic glycolysis (Figure 1). It was previously demonstrated that MIF induces the synthesis of fructose 2,6-bisphosphate (F2,6BP) by activating the phosphofructo-2-kinase/fructose-2,6-bisphosphatase (PFK-2) bifunctional enzyme in rat myotubes in vitro [95]. Moreover, it was also shown that LPS-activated monocytes express an inducible form of PFK-2 and synthesize elevated levels of F2,6BP [96], an allosteric activator of glycolysis, which stimulates lactate production, leading to increased ECAR [95]. Thus, we hypothesized that PFK-2 may be one of the potential molecular targets of MIF during the regulation of glycolysis in RAW264.7 macrophages. In our hands, LPS+IFN-γ most likely stimulated MIF, which than can upregulate glycolysis by enhancing F2,6BP production. Contrary to the above, KRP-6 may prevent F2,6BP production, thereby suppressing ECAR (Figure 6A,B), which reflects lactate synthesis and the rate of aerobic glycolysis. The hypothesis, which implies KRP-6 regulates glycolysis in macrophages, was further strengthened by our findings, in which F_O_F_1_-ATPase inhibitor oligomycin improved ECAR in the VEH group, yet failed to do so in the LPS+IFN-γ+KRP-6 group (Figure 6A,C). Previously, oligomycin was shown to induce F2,6BP production in activated monocytes [96]. In our experiments, oligomycin may induce F2,6BP production in VEH cells; however, was unable of further activation in LPS+IFN-γ+KRP-6-treated cells. This occurred likely due to KRP-6 inhibited MIF, PFK-2 was probably blocked, and the blockade could not be lifted by oligomycin. Surprisingly, in the LPS+IFN-γ group, oligomycin inhibited ECAR (Figure 6C). Clarification regarding this phenomenon requires further investigation. The suppressed glycolytic rate in LPS+IFN-γ+KRP-6-treated cells persisted, even following FCCP, and rotenone + antimycin A treatment when compared to VEH or LPS+IFN-γ groups (Figure 6A).

Since KRP-6 effectively inhibited macrophage activation [97,98] and reduced IFN-γ-induced ROS production without a radical scavenging activity (Figure 5A,B), we hypothesized MIF-mediated ROS [99] may be one of the molecular initiators of metabolic reprograming in macrophages. Knowingly, ROS is capable of inhibiting the complexes of the respiratory chain [100] and F_O_F_1_-ATPase activity [100,101]. We discovered that the F_O_F_1_-ATPase inhibitor oligomycin did not reduce OCR in LPS+IFN-γ-treated cells, yet resulted in a significant decrease in our LPS+IFN-γ+KRP-6 treated group (Figure 7B). An explanation for the findings may be that ROS entirely abolished the activity of the respiratory chain and F_O_F_1_-ATPase, which could not be further decreased by oligomycin. In contrast to these results, KRP-6 reduced MIF-induced ROS, which resulted in more healthy coupled mitochondria in which oligomycin effectively reduced OCR by blocking F_O_F_1_-ATPase. In addition, OCR increased in our LPS+IFN-γ+KRP-6 group following FCCP treatment, while it remained unaltered in LPS+IFN-γ-treated cells (Figure 7B). FCCP is a mitochondrial uncoupler, which separates the activity of the respiratory chain from F_O_F_1_-ATPase [102]. In LPS+IFN-γ-treated cells, the respiratory chain was entirely corrupted by ROS, thus OCR did not increase, even following FCCP treatment. In contrast, the uncoupler FCCP liberated the respiratory chain in LPS+IFN-γ+KRP-6-treated cells, which resulted in an elevated OCR, since complexes of the respiratory chain remained more integral and functional (Figure 7B). However, the finding in which OCR in the FCCP-treated LPS+IFN-γ+KRP-6 group did not reach the level of OCR in the FCCP-treated VEH cells indicated that the respiratory chain remained still partially inhibited. Accordingly, MIF-induced ROS may not be the only mechanism, yet is a critical mechanism by which macrophages downregulate mitochondrial energy production (Figure 7B).

Knowingly, LPS-activated M1 macrophages downregulate PARP-1 expression [29]. Our findings, in which KRP-6 improved PARP-1 and PARP-2 transcription in LPS+IFN-γ-induced cells, indicate a regulatory role for MIF regarding this process.

## 5. Conclusions

In conclusion, in our present study, we utilized KRP-6, a highly selective MIF ketonase inhibitor, to investigate its effects on classical M1 macrophage polarization. We revealed that KRP-6 inhibited ROS production, reduced glycolytic flux, and improved mitochondrial energy production. Additionally, KRP-6 also upregulated PARP-1 and PARP-2 transcription, indicating a regulatory role for MIF during PARP transcription. To determine whether these processes are MIF receptor-mediated or MIF tautomerase-activated is beyond the scope of this study. Nonetheless, to separate the biological consequences of receptor activation and tautomerase activity, and to specifically identify the processes dependent only on ketonase or enolase activity, is of immense interest. Since proline-1 mutant MIFs lost tautomerase activity, a genetically modified MIF is not suitable for investigating ketonase and enolase activities separately. For this purpose, the application of selective inhibitors may become a potential solution. Here, we demonstrated that KRP-6, a highly selective ketonase inhibitor, significantly reduces macrophage activation and leukocyte migration, thus it may represent a promising pharmacotherapeutic approach to treating or aiding in numerous chronic inflammatory and autoimmune diseases. Since the anti-inflammatory effect of KRP-6 was only investigated in cell cultures, the clinical application of KRP-6 requires further studies. In addition, together with other potent and highly selective inhibitor molecules, KRP-6 may prove beneficial in clarifying the exact and separate roles of MIF ketonase and enolase, thus providing new insights into MIF biology.

## Figures and Tables

**Table 1:** Primers for RT-PCR analysis.

Accession NM	Name	Sequence (5′-3′) (from Integrated DNA Technologies, BVBA, Belgium)	Amplicon Size (bp)
NM_007415	PARP1	F-GAGTACAGTGCCAGTCAGC	117
		R-CACCTCGTCACCTTTTCTCTT	
NM_009632	PARP2	F-GTGGACCCAGAGTGTGCAGCC	194
		R-CCCGTCTTTCCAACTCGGCCC	
NM_145619	PARP3	F-TGCTGCTGGTGCTAGCGGAC	281
		R-GCCCAGTTTGGAGTGGGCCTG	
NM_008084	GAPDH	F-AATGGTGAAGGTCGGTGTG	150

## Data Availability

The data presented in this study are available in the current article.

## Supplementary Materials

The following supporting information can be downloaded at: https://www.mdpi.com/article/10.3390/antiox12101790/s1, Figure S1: Chemotaxis gating strategy.
