# Supplementary material for: Highly Selective MIF Ketonase Inhibitor KRP-6 Diminishes M1 Macrophage Polarization and Metabolic Reprogramming
Source: Antioxidants (Basel). 2023 Sep 22;12(10):1790. doi: 10.3390/antiox12101790 (PMC10604361; doi:10.3390/antiox12101790)
Supplement: Supplementary file 1 [file antioxidants-12-01790-s001.zip › antioxidants-2554563-supplementary.pdf]

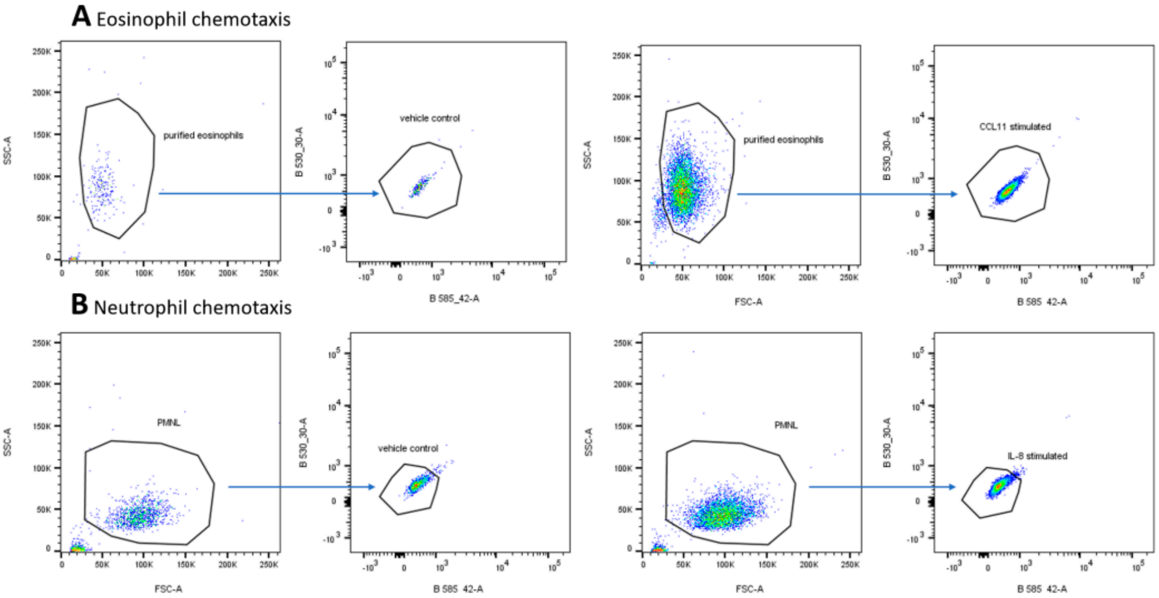

Figure S1. Chemotaxis gating strategy. A) Purified eosinophils or B) Polymorphonuclear leukocytes (PMNL) were allowed to migrate towards respective chemoattractants (i.e. CCL11 or IL-8) in a microBoyden chamber at 37 °C for 60 min. Migrated cells were enumerated by flow cytometry on a BD Canto II flow cytometer (acquisition set for 30 sec at medium flow rate). As shown, eosinophils and neutrophils were gated by their forward and side scatter properties and by autofluorescence.
